# Effects of drought-stress on seed germination and growth physiology of quinclorac-resistant *Echinochloa crusgalli*

**DOI:** 10.1371/journal.pone.0214480

**Published:** 2019-04-04

**Authors:** La-Mei Wu, Yong Fang, Hao-Na Yang, Lian-Yang Bai

**Affiliations:** 1 Long Ping Branch of Graduate School, Central South University, Changsha, Hunan, China; 2 Hunan Agricultural Biotechnology Research Institute, Hunan Academy of Agricultural Sciences, Changsha, Hunan, China; Estacion Experimental del Zaidin, SPAIN

## Abstract

*Echinochloa crusgalli* (L.) Beauv. (barnyard grass) is considered a noxious weed worldwide, and is the most pernicious weed decreasing rice yields in China. Recently, *E*. *crusgalli* has evolved quinclorac resistance, making it among the most serious herbicide resistant weeds in China. The present study explored differences in germination and growth between quinclorac-resistant and -susceptible *E*. *crusgalli* collected in Hunan Province. The order of the seven *E*. *crusgalli* biotypes assessed, from high to low quinclorac-resistance, was: quinclorac-resistant, Chunhua, Hanshou, Shimen, Hekou, Dingcheng, and quinclorac-susceptible. With an increased in the level of quinclorac-resistance, the germination rate, length of young shoots and roots, and fresh weight of *E*. *crusgalli* were all decreased compared with that in more susceptible biotypes. However, there were no significant differences between quinclorac-resistant and susceptible *E*. *crusgalli* biotypes without polyethylene glycol 6000 treatment. Drought had a more obvious effect on glutathione S-transferases (GST) activity, determined by spectrophotometric method, in quinclorac-resistant *E*. *crusgalli*. Higher resistance level biotypes showed greater activity, and when treated with polyethylene glycol 6000 for 3 days, all *E*. *crusgalli* biotypes showed the highest GST activity. This study demonstrated that as the level of quinclorac-resistance increased, the rate of seed germination decreased, while the growth of young buds, young roots, and fresh weight decreased. Increased quinclorac-resistance may be related to the increased metabolic activity of GST in *E*. *crusgalli*.

## Introduction

*Echinochloa crusgalli* (L.) Beauv. (barnyardgrass), a C4 plant [1], is regarded as the most dominant and troublesome weed in rice fields [2]. It competes with rice for water, light, and soil nutrients [3,4] and has a severe impact on rice yield and quality [5]. *Echinochloa crusgalli* is also one of the most common weeds found in Chinese farmland [6]. In addition, it displays strong ecological adaptability. For example, its seeds can germinate under an anaerobic environment, germinate and mature rapidly, and be produced in large numbers [7,8].

Quinclorac, belonging to the class of quinolinecarboxylic acid, is one of the auxinic herbicides. It can effectively control annual grasses in rice fields, including *E*. *crusgalli*. However, its extensive and continuous use, often as the sole method of weed control, has decreased the sensitivity of *E*. *crusgalli* to quinclorac [9–13]. Due to its excellent efficacy in controlling *E*. *crusgalli*, quinclorac has been extensively used in Chinese rice fields since the 1990s [14]. Quinclorac-resistant *E*. *crusgalli* not only reduces rice yield but also seriously damages the farmland ecosystem [15,16], presenting considerable challenges to weed management in rice fields. In addition, auxinic herbicides may possess potential risks to humans and the environment [17].

Successful establishment of weeds depends heavily on their ability to evolve stress tolerance, and weeds tend to exhibit a increasing tolerace to stress than crops [18]. Plants improve their adaptability to thrive in arid environments mainly by evolving several mechanisms of drought escape, tolerance, and avoidance [19]. Different plants show varying tolerance levels to drought stress. These differences are mainly reflected in plant weight (vegetative growth) and number of seeds (reproductive growth) [20–23]. In most cases, weeds are more tolerant to drought than crops, leading to reduced crop and increased weeds in drought years [24]. Plant growth is affected under prolonged drought conditions; for example, the leaf biomass and growth rate of *Archontophoenix cunninghamiana* decreases in dry conditions [25]. Sushila *et Al*. reported that tomato plant height decreased under two drought-stress treatments (3 days of drought-stress before metribuzin application with no drought-stress after application, and 3 days of drought-stress before metribuzin application with 3 d of drought-stress after application) [26].

The response of plants to drought stress is related to many factors, including Glutathione S-transferases (GSTs, EC 2.5.1.18) activity and expression. Gallé et al. researched GST activity and expression patterns during grain filling in flag leaves of wheat genotypes differing in drought tolerance. They observed high GPOX activity (EC 1.11.1.9) in well-watered controls of a cultivar of the drought-tolerant Plainsman and expression of six GST genes inducing by drought stress in all of the four investigated cultivars [27]. However, there is very limited information about the effect and mechanism of drought stress on the growth of herbicide-resistant weeds. Therefore, it is necessary to identify the reaction of herbicides-resistant weeds at germination and early growth stages for successful weed management in rice fields under drought environment.

## Materials and methods

### Specimens

*Echinochloa crusgalli* seeds used in this study were collected in 2013 from two adjacent rice fields in Chunhua, Hunan Province (*E*. *crusgalli* is a very common weed that occurs in paddy fields, it exists in every paddy field in Hunan Province, and the researchers collect only *E*. *crusgalli* seeds or whole plants in paddy field never need the permission by the authority. And the owner of the land welcome our researches which will help them for solving the problem of quinclorac-resistant *E*. *crusgalli*, as long as our researchers do not destroy the rice in the field. Beyond that, we just collected the seeds of *E*. *crusgalli*, and we promise this study did not involve endangered or protected species). In field A (N 28°18’21”, E 113°17’21”), quinclorac (1200 g a.i./ha) had failed to control *E*. *crusgalli* after more than 15 years of use, while field B (N 28°17’37”, E 113°15’57”) had not received quinclorac application in the previous 5 years. The collected *E*. *crusgalli* seeds from field A and B were cultivated to tillering in plastic pots (30 cm×20 cm×12 cm). Two tillers were collected from the same plant: one for the sensitivity analysis and the other for seed collection. One tiller was treated with quinclorac (400 g a.i./ha) for determining quinclorac-resistance, and the other tiller was used for collecting seeds. Moreover, 21 days after quinclorac treatment, the *E*. *crusgalli* plants of biotype B were all dead, while those of biotype A were all alive. The surviving tillers were considered resistant, while the dead tillers were considered susceptible.

The susceptible and resistant seeds were all preserved. Seed propagation was conducted in a breeding pool for three generations from 2014 to 2016. In the fourth generation, the quinclorac-resistant biotype was named RR, and the quinclorac-susceptible biotype was named SS. The other five *E*. *crusgalli* biotypes were collected in 2016 from different regions in Hunan Province: Dingcheng (DC N 28°55’6”, E 111°45’18”), Hanshou (HS,N 29°6’31”, E 111°53’26”), Shimen (SM, N 29°38’38”, E 111°11’45”), Hekou (HK, N 27°42’18”, E 112°53’47”) and Chunhua (CH, N 28°18’21”, E 113°17’21”). All of these biotypes had a history of quinclorac application.

Quinclorac (96%) was provided by Jiangsu Tianrong Corporation (LS20053491), and polyethylene glycol-6000 (PEG-6000) was purchased from Shanghai Chemical Reagent (20130918).

### Detection of quinclorac-resistance level for RR, SS and the five *E*. *crusgalli* Biotypes

*Echinochloa crusgalli* seeds were selected and soaked in 0.5% KNO_3_ for 24 hours to break dormancy. The seeds were then washed with water, sown in a plastic pot with a diameter of 10 cm and a height of 6 cm, and cultivated at 28°C to accelerate germination. When the seedlings reached the 3-5-leaf stage, quinclorac was sprayed by a spray tower (3WP-2000, Nanjing, China), and applied at 0, 200, 400, 800, 1,600, and 3,200 g a.i./ha to RR and the CH biotype, and at 12.5, 25, 50, 100, and 200 g a.i./ha to SS and the other four biotypes. Three weeks after treatment, all plants were cut at the soil surface and the fresh weight for each pot was determined. All experiments were conducted in a completely randomized design with three replications, and each experiment was conducted twice. The rate of fresh weight inhibition and the relative resistance index were calculated as follows:

Fresh weight inhibition rate (%) = 100% × (fresh weight of control—fresh weight of treatment) / fresh weight of control

Relative resistance index (R/S) = Resistance GR_50_ / Susceptible GR_50_

### Effect of PEG-6000 simulated drought stress on *E*. *crusgalli* seed germination

Whole, healthy seeds were selected and dormancy was broken as above. Then, the seeds were air dried. One hundred dry seeds were placed in a 9-cm-diameter Petri dish containing two layers of Whatman filter paper moistened with 10 ml of deionized water or PEG-6000 solution [28]. The petri dish was composed of 3-mm-thick glass with good sealing, which minimized evaporation. The six PEG-6000 concentrations were 0, 1%, 5%, 10%, 15%, and 20%. A completely randomized design with three replications was applied to all of the experiments. Seed germination was investigated at 48 hours, 72 hours and 96 hours after treatment.

### Effect of PEG-6000 simulated drought stress on the growth of *E*. *crusgalli* young shoots and roots

Seeds were treated and the six concentrations of PEG-6000 solution were applied as described above. Twenty germinated seeds were chosen and cultivated at 28°C, Each experiment was repeated three times. The length of the young shoots and roots were measured in the various treatments, and the inhibition rates were calculated after 4 and 7 days of treatment as follows:

Inhibition rate (%) = 100% × (length of young shoots or roots of control—length of young shoots or roots of treatment) / length of young shoots or roots of control

### Effect of PEG-6000 simulated drought stress on the fresh weight of *E*. *crusgalli*

The plants were treated with PEG-6000 when the young buds of *E*. *crusgalli* grew to 2–3 cm. Hoagland's solution was prepared for plant growth. A certain amount of PEG-6000 was dissolved in the Hoagland's solution, and the PEG-6000 concentrations were 1%, 5%, 10%, 15%, and 20%. Following this, 3.5 L of Hoagland's solution (containing PEG-6000) was poured into a 4-L plastic box (25 cm x 16 cm x 10 cm). The top of the plastic box was covered with a foam plate with 10 holes, and the top was wrapped with gauze, ensuring that the foam plate completely came in contact with the nutrient solution [29]. The seedlings were transplanted to the small holes in the foam plate, and two plants fit into one hole. After 21 days, the fresh weight was investigated in the various treatments, and the fresh weight inhibition rate was calculated as described above.

#### GST activity of *E*. *crusgalli* under drought stress

The plants were cultivated in Hoagland's solution. When plants grew to three to four leaves, they were treated with 15% PEG-6000 as described above. The samples were collected at 0, 1, 3, 5 and 14 days. The fresh leaves were removed and weighted, then wrapped with silver paper and cooed with liquid nitrogen. They were stored in a refrigerator at -80°C. Tissue extracts were prepared by homogenizing leaf tissue (three-leaf stage) in liquid nitrogen. The homogenate was then centrifuged for 20 min at 12000 g and filtered. A GST assay kit (Nanjing Jiancheng Bioengineering Istitute, Jiangsu, China, A004) was used, and protein content was determined by the method of Bradford [30]. GSH formation was recorded at 28°C on a spectrophotometer 721 (INESA, Shanghai, China) at 412 nm.

### Statistical analysis

Analysis of variance was performed using Duncan's multiple-range test to compare treatment means. Pearson correlation coefficients were calculated using SPSS 10.0, and figures were drawn using Sigma Plot 10.0.

## Results and discussion

### Susceptibility of *E*. *crusgalli* to quinclorac

Quinclorac-resistant *E*. *crusgalli* was first confirmed in Hunan Province in 2002 [31]. Some other resistant populations were then subsequently found [32,33]. Quinclorac-resistance level increases with the amount and duration of quinclorac application. This also might be caused by the unscientific and inappropriate use of quinclorac, leading to a decrease in the susceptibility to the agent and a corresponding increase in the risk of herbicide resistance [34,35]. In the present study, a large difference in quinclorac resistance was observed among the seven *E*. *crusgalli* biotypes (Table 1). Compared with the SS biotype with a GR_50_ of 22.38 g a.i./ha, the RR and CH biotypes were not susceptible to quinclorac, and the GR_50_ of RR and CH (3106.94 g. a.i./ha and 2685.92 g. a.i./ha, respectively) was much higher than the recommended field dose(225–375 g. a.i./ha) in China. Based on the resistance index, RR populations had GR_50_ values 138.86-fold higher than that of SS, While that of the CH biotype was 120.01-fold higher; the relative resistance index values of the HS, SM, HK, and DC biotypes were 5.09, 2.81, 1.83, and 1.07, respectively. Therefore, the order of the seven *E*. *crusgalli* biotypes, from high to low quinclorac-resistance was: RR, CH, HS, SM, HK, DC, and SS. Both resistant and susceptible *E*. *crusgalli* are harmful to crops. Global losses of rice yield due to *E*. *crusgalli* competition (by removing up to 80% of the available soil N_2_), are estimated to be approximatelty 35% [36]. The high level of nitrate accumulation is harmful to livestock. *Echinochloa crusgalli* also acts as a host for several mosaic virus diseases [37].

**Table 1:** Quinclorac dose–response analyses for *E*. *crusgalli* biotypes.

Weed populations	Toxicity regression equations Y = a + b x	GR_50_ (g a.i./ha)	GR_90_ (g a.i./ha)	Resistant index
RR	Y = 0.5826 + 1.2649 x	3106.94 (2753.97–3505.15)	32026.65 (24325.24–42166.33)	138.86
CH	Y = 0.9263 + 1.1880 x	2685.92 (2480.41–2908.45)	32199.95 (26487.52–39144.35)	120.01
HS	Y = 2.4026 + 1.2630 x	113.89 (84.14–154.15)	1177.98 (538.71–2075.84)	5.09
SM	Y = 2.9095 + 1.1625 x	62.85 (58.58–67.42)	795.59 (648.24–976.44)	2.81
HK	Y = 2.7845 + 1.3734 x	41.03 (34.56–48.71)	351.75 (241.86–511.55)	1.83
DC	Y = 2.9026 + 1.5216 x	23.90 (20.08–25.87)	166.19 (150.34–183.71)	1.07
SS	Y = 2.5854 + 1.7889 x	22.38 (21.55–23.23)	116.46 (112.08–121.00)	1

### Effect of PEG-6000 simulated drought stress on *E*. *crusgalli* seed germination

**Fig 1:**
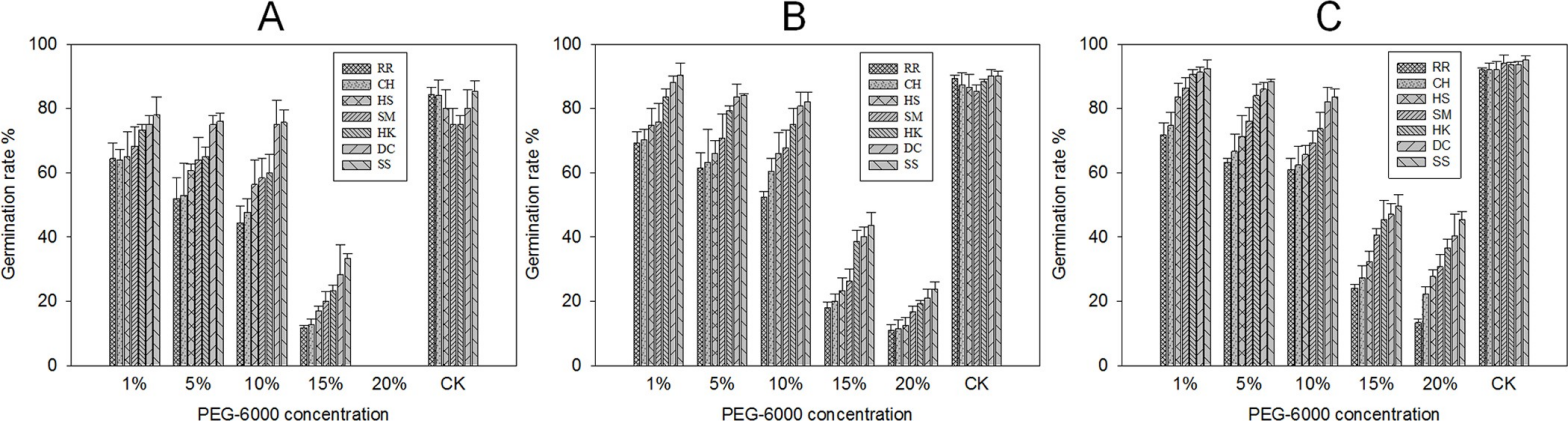
Germination rate (%) of the seven *E*. *crusgalli* biotypes under five PEG-6000 concentrations at different time periods. *E*. *crusgalli* germination rate (%) under five PEG-6000 concentrations at (A) 48 hours, (B) 72 hours, and (C) 96 hours of treatment. Data are expressed as least square means ± standard error.

Drought is an important factor that negatively affects plant growth. PEG-6000 was used to induce drought stress as it can modify the osmotic potential of nutrient solution cultures [38]. In the present study, drought-stress inhibited *E*. *crusgalli* seed germination to a certain extent. At the same PEG-6000 concentration, the six other biotypes had a slower germination rate compared with the SS biotype. As the PEG-6000 content increased, the germination rates decreased. *E*. *crusgalli* seeds showed good germination with PEG-6000 concentrations below 10% (Fig 1). When the concentration of PEG-6000 exceeded 15%, the germination rates decreased sharply, and none of the biotypes germinated when the PEG-6000 concentration was 20%. As the *E*. *crusgalli* resistance level incresed, the germination rate decreased at the same PEG-6000 concentrations. In addition, the germination rate at 7 days was higher than that at 4 days post treatment, while there was no significant difference among various resistant levels in plants not treated with drought-stress. Drought stress is one factor that can affect the germination of resistant and susceptible weeds. Some plants even present differences between resistant and susceptible types[19,39]. Numerous studies have showed that glyphosate-resistant plants produce fewer but larger seeds, while glyphosate-susceptible plants produce a greater number of smaller seeds [40].

### Effect of PEG-6000 simulated drought stress on *E*. *crusgalli* shoot and root growth

**Fig 2:**
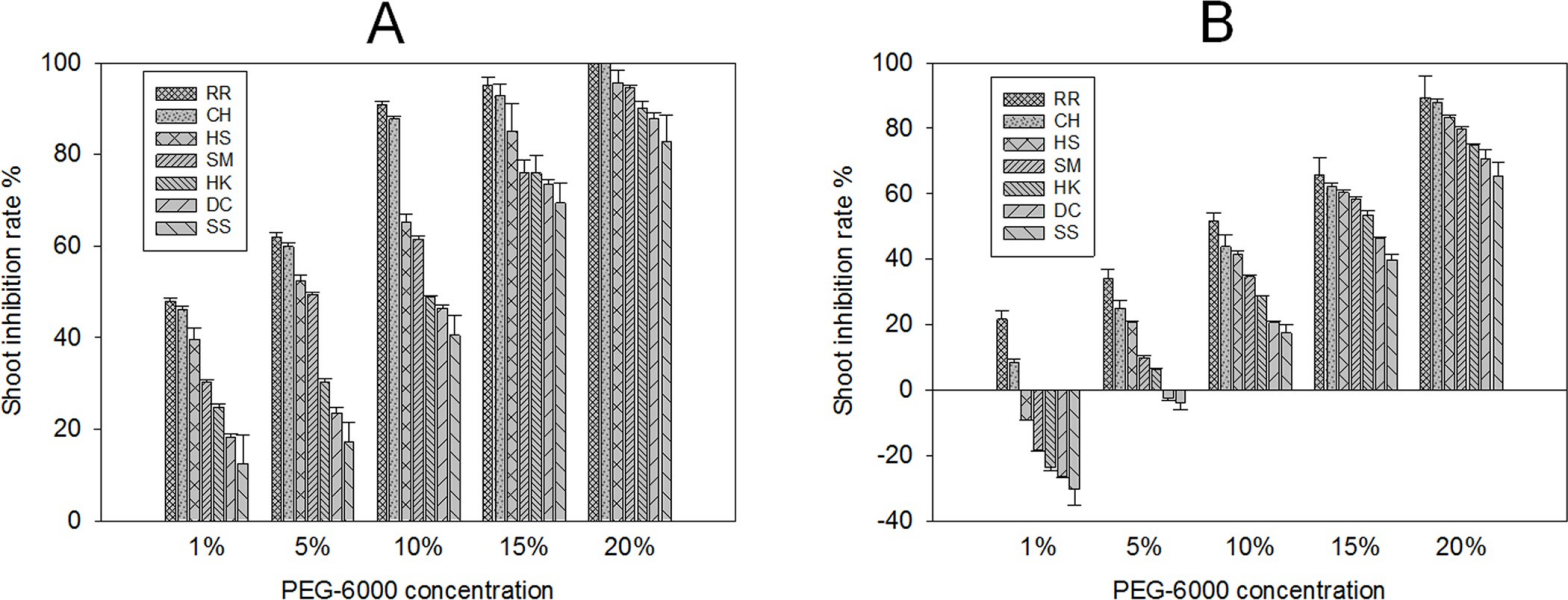
*E*. *crusgalli* shoot inhibition rate (%) under five PEG-6000 concentrations at different time periods. *E*. *crusgalli* shoot inhibition rate (%) under five PEG-6000 concentrations at 4 days (A) and 7 days (B) of treatment. Data are expressed as least square means ± standard error.

**Fig 3:**
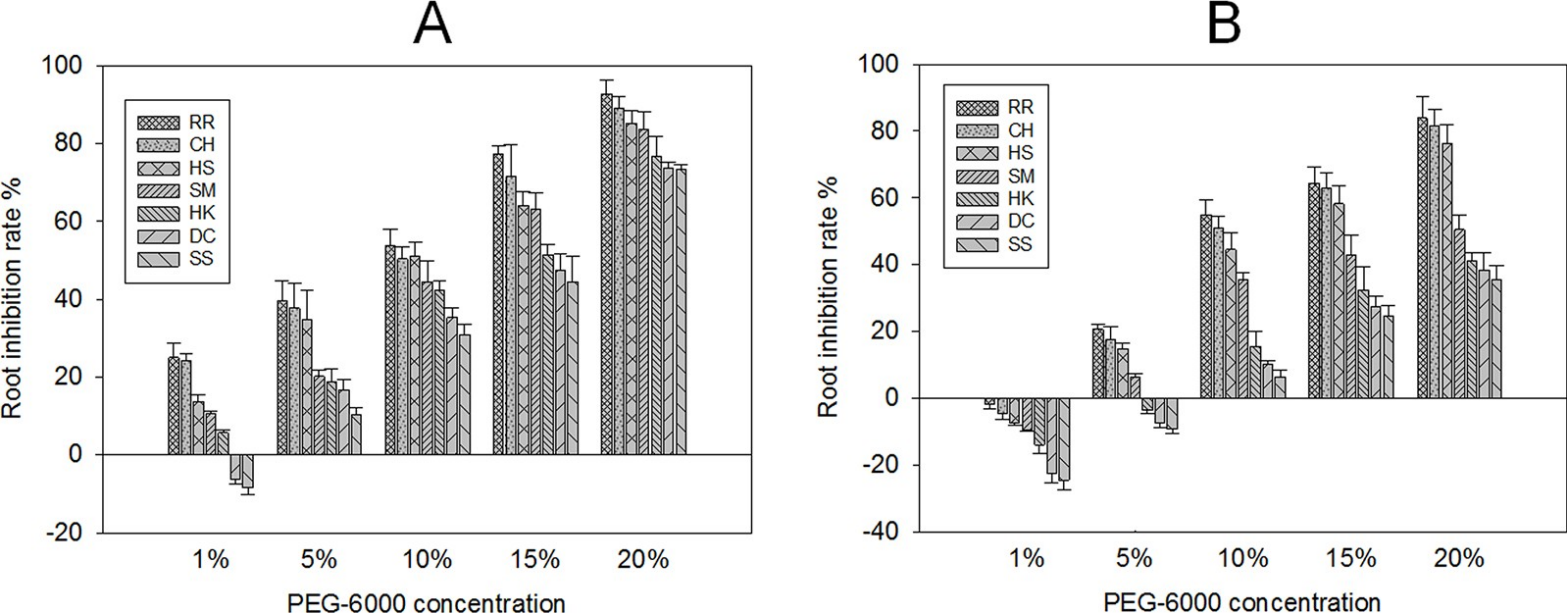
*E*. *crusgalli* root inhibition rate (%) under five PEG-6000 concentrations at different time periods. *E*. *crusgalli* root inhibition rate (%) under five PEG-6000 concentrations at 4 days (A) and 7 days (B) of treatment. Data are expressed as least square means ±standard error.

Drought stress significantly reduces stalk diameter, root length, root surface area, root volume and root dry weight [41]. It can affect not only the elongation of young shoots and roots, but also plant shape. In response to drought, root hairs become bulbous and are shortened, even tuberized, and hairless roots can form in soil-grown Arabidopsis [42,43]. In the present study, drought-stress affected *E*. *crusgalli* shoot and root growth. At the same PEG-6000 concentration, compared with the SS biotype, the growth inhibition rate of young germinated buds and roots of the other six biotype was higher. With increasing PEG-6000 content, the growth inhibition rate of all seven biotypes increased. At 4 days after treatment (Fig 2A, Fig 3A), the growth of young shoots and roots of *E*. *crusgalli* were all inhibited by 1%-20% PEG-6000, except the roots of the DC and SS biotypes. However, 7 days after treatment (Fig 2B, Fig 3B), 1% PEG-6000 accelerated shoot and root growth of sensitive biotypes but inhibited the shoot growth of resistant biotypes, similar to cotton [28] and canola [44] cultivated under drought stress. With increasing quinclorac-resistance level, PEG-6000 had a more obvious inhibitory effect on the growth of the young shoots and roots of *E*. *crusgalli*. Shoot and root growth was severely inhibited by 20% PEG-6000, which was one of the most resistant to quinclorac. There was no significant difference in shoot and root growht among the seven biotypes without PEG-6000 treatment.

### Effect of PEG-6000 simulated drought stress on *E*. *crusgalli* fresh weight

**Fig 4:**
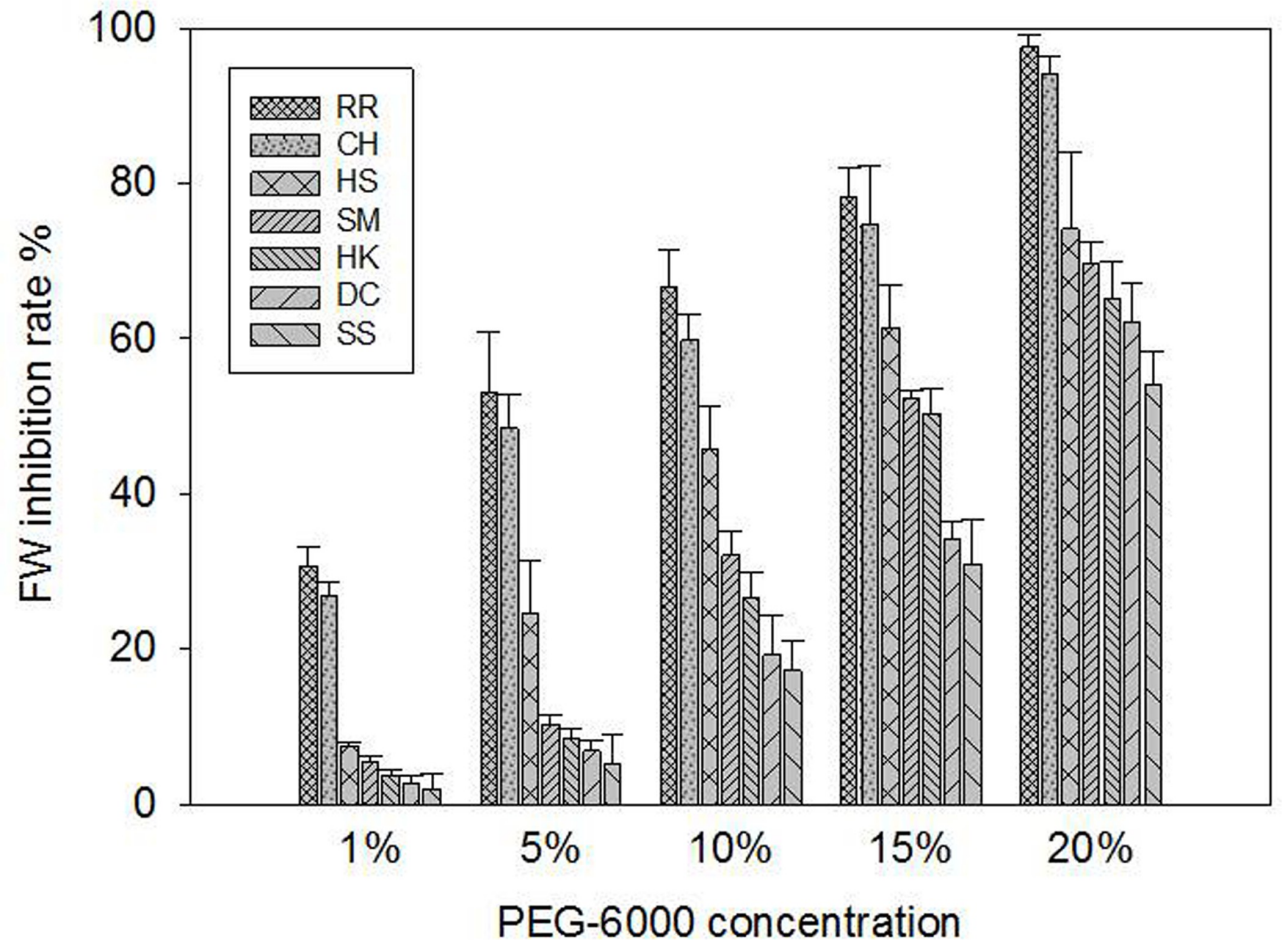
*E*. *crusgalli* fresh weight (FW) inhibiton rate (%) under five PEG-6000 concentrations 21 days after treatment. Data are expressed as least square means ± standard error.

Drought stress reduces the efficiency of N_2_ fertilization, significantly decreasing plant height, total leaf area, root and shoot dry biomass, dry matter accumulation, and yield [28,45]. Terminal drought stress (drought at the reproductive growth stage) substantially reduces growth performance in terms of reduced leaf area index, crop growth rate, net assimilation rate, and total dry matter production [36]. Other stressors such as salinity and salicylic acid can influence the response of plants to herbicides by regulating physiological and biochemical reactions [41,46]. Under quinclorac stress, salicylic acid enhanced the expression of some genes to accelerate the metabolism of quinclorac for the protection of rice [45]. In the present study, drought stress reduced the fresh weight of seven *E*. *crusgalli* biotypes to different degrees. At the same PEG-6000 concentration, compared with the SS biotype, the fresh weight inhibition rate of the other six biotypes was higher. With increasing PEG-6000 concentrations, the inhibition rate of fresh weight increased obviously. The fresh weight of *E*. *crusgalli* of higher quinclorac-resistance level was inhibited more than the sensitive biotypes. Inparticular, in the RR and CH biotypes, which were the most resistant to quinclorac, the fresh weight inhibition rate reached up to 94% when the concentration of PEG-6000 was 20%, while for the other five biotypes this was 54%–74% (Fig 4).

### Effect of PEG-6000 simulated drought stress on *E*. *crusgalli* GST Activity

**Fig 5:**
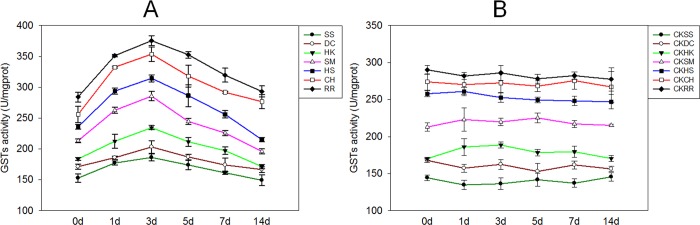
GST activity of *E*. *crusgalli* under 15% PEG-6000 treatment. (A) GST activity of *E*. *crusgalli* under 15% PEG-6000 treatment. (B) GST activity of *E*. *crusgalli* without PEG-6000 treatment. Data are expressed as least square means ± standard error.

Plant GSTs have been proposed to afford protection under various stress conditions and they were known to function in herbicide detoxification, hormone homeostasis, vacuolar sequestration of anthocyanin, tyrosine metabolism, hydroxyperoxide detoxification, regulation of apoptosis, and in plant responses to biotic and abiotic stress [47]. In the present study, enzymatic activity in E. crusgalli seedlings was determined using a GST assay kit; this is a conventional technique based on spectrophotometric measurements with the model GST substrate CDNB. This compound is used for detecting GST in all organisms [48]. Results showed that under 15% PEG-6000 treatment, the SS biotype which was the most sensitive to quinclorac, had the highest GST activity, especially after 3 days of treatment when the GST activity was 375 U/mgprot. The CH biotype which was the most resistant to quinclorac, had the lowest GST activity among the seven biotypes. The order of the seven *E*. *crusgalli* biotypes from low to high GST activity was: RR, CH, SH, SM, HK, DC, and SS. GST activity of all seven biotypes initially increased and then subsequently decreased (Fig 5).

## Conclusions

This study found that the germination and seedling growth of quinclorac-resistant *E*. *crusgalli* biotypes are more sensitive under drought stress. In the CH population, the resistance level of quinclorac was the highest, which is mainly the result of 15 years of quinclorac application. In the present study, the HS, HK, and DC populations all showed different levels of quinclorac resistacne due to the varying levels of quinclorac application. Drought stress not only inhibited germination and growth of quinclorac-resistant *E*. *crusgalli*, but also significantly inhibited their GST activity, which lead to poorer adaptability of quinclorac-resistant *E*. *crusgalli* under drought stress. The present research indicates that increased quinclorac-resistance may be related to the increased metabolic activity of GST in *E*. *crusgalli*. This information can provide a scientific basis for understanding the occurrence of quinclorac-resistant *E*. *crusgalli*, and provide key information for the management of resistant weeds. Therefore, these data are valuable for increasing agricultural production and farmer incomes. The findings revealed that the formation and development of quinclorac-resistance in *E*. *crusgalli* may reduce its adaptability to drought, and with the exception of applying other herbicides, regulating the soil drought condition may represent a method for managing the quinclorac resistance of *E*. *crusgalli*.
